# Bis{5-meth­oxy-2-[(1*H*-pyrrol-2-yl)methyl­imino­meth­yl]phenolato}zinc(II)

**DOI:** 10.1107/S1600536809032784

**Published:** 2009-08-22

**Authors:** Ying-Jie Cai, Peng Huang, Jin Li, Qiang Wang

**Affiliations:** a Engineering Research Center for Clean Production of Textile Dyeing and Printing, Ministry of Education, Wuhan 430073, People’s Republic of China

## Abstract

The title compound, [Zn(C_13_H_13_N_2_O_2_)_2_],  contains a Zn(II) centre, located on a twofold rotation axis, that is coordinated by two O atoms and two N atoms from two salicylal Schiff base mol­ecules. The crystal structure is stabilized by inter­molecular C—H⋯O hydrogen bonds.

## Related literature

For the importance of zinc derivatives in biological processes, see: Chen *et al.* (2007 ▶); Xiao & Xiao (2008 ▶); Xiao *et al.* (2007 ▶, 2008 ▶). For related structures, see: You & Zhu (2006 ▶); Zhu *et al.* (2004 ▶); Qiu *et al.* (2004 ▶).
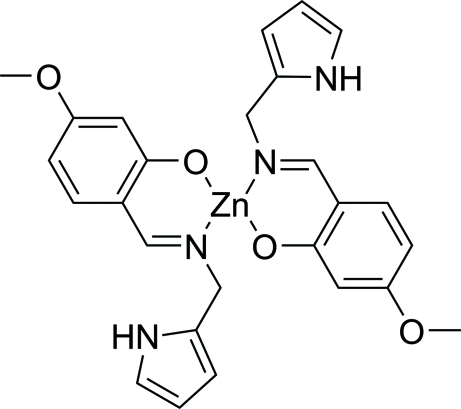


## Experimental

### 

#### Crystal data

[Zn(C_13_H_13_N_2_O_2_)_2_]
*M* *_r_* = 523.88
Monoclinic, 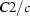

*a* = 27.210 (4) Å
*b* = 5.2239 (9) Å
*c* = 24.335 (3) Å
β = 137.179 (9)°
*V* = 2351.1 (6) Å^3^
*Z* = 4
Mo *K*α radiation
μ = 1.09 mm^−1^
*T* = 298 K
0.30 × 0.30 × 0.20 mm

#### Data collection

Bruker SMART APEX area-detector diffractometer
Absorption correction: multi-scan (*SADABS*; Sheldrick, 1996 ▶) *T*_min_ = 0.736, *T*_max_ = 0.812
8100 measured reflections
2881 independent reflections
2429 reflections with *I* > 2σ(*I*)
*R*_int_ = 0.028

#### Refinement

*R*[*F* ^2^ > 2σ(*F* ^2^)] = 0.058
*wR*(*F* ^2^) = 0.154
*S* = 1.08
2881 reflections
160 parameters
H-atom parameters constrained
Δρ_max_ = 0.72 e Å^−3^
Δρ_min_ = −0.57 e Å^−3^

### 

Data collection: *SMART* (Bruker, 2000 ▶); cell refinement: *SAINT* (Bruker, 2000 ▶); data reduction: *SAINT*; program(s) used to solve structure: *SHELXS97* (Sheldrick, 2008 ▶); program(s) used to refine structure: *SHELXL97* (Sheldrick, 2008 ▶); molecular graphics: *SHELXTL* (Sheldrick, 2008 ▶); software used to prepare material for publication: *SHELXTL*.

## Supplementary Material

Crystal structure: contains datablocks global, I. DOI: 10.1107/S1600536809032784/hg2551sup1.cif

Structure factors: contains datablocks I. DOI: 10.1107/S1600536809032784/hg2551Isup2.hkl

Additional supplementary materials:  crystallographic information; 3D view; checkCIF report

## Figures and Tables

**Table 1:** Hydrogen-bond geometry (Å, °)

*D*—H⋯*A*	*D*—H	H⋯*A*	*D*⋯*A*	*D*—H⋯*A*
C9—H9*A*⋯O1^i^	0.97	2.32	3.291 (4)	177

Symmetry code: (i) 
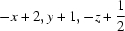
.

**Fig. 1.**
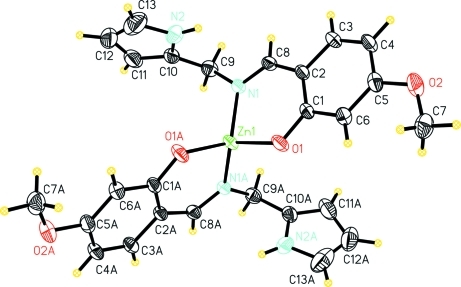
The independent components of (I), showing the atom-labelling scheme. Displacement ellipsoids are drawn at the 30% probability level.

## supplementary crystallographic information

### Comment

Zinc derivatives are particularly interesting owing to their essential importance in several biological processes (Xiao *et al.*, 2008; Xiao & Xiao, 2008; Xiao *et al.*,  2007; Chen *et al.*, 2007). We have reported the structures of a few zinc(II) complexes (You & Zhu, 2006; Qiu *et al.*, 2004; Zhu *et al.*, 2004). As an extension of our work on the structural characterization of zinc compounds, we report the crystal structure of the title compound, (I), which has been determined in an attempt to understand the structural behaviour of nitrogen containing ligands.

The present X-ray single-crystal diffraction study reveals that compound (I), Bis(4-methoxysalicylidene(1*H*-pyrrol-2-yl)methanaminato)zinc(II), consists of a Zn(II) atom and two bidentate salicylal Schiff base ligands. As shown in Fig. 1, The central Zn atom exhibits 4-coordination by two N atoms from imine moieties and two O-anions from salicylal groups, forming a slightly distorted tetrahedron. The distortion arises from the difference between O—Zn and Zn—N (Table 1). The O1—C1 distance (1.308 (3) Å) and C2—C8 (1.425 (4) Å) are shorter than classical single C—O and C—C bonds, respectively. This suggests that electron significantly delocalized over the O1—C1—C2—C8—N1 group and the same to the O1A—C1A—C2A—C8A—N1A group. Zn1, N1, C8 and O1 almost stand in the plane of ring C1 to C6, and makes dihedral angle of 85.569 (45) ° with corresponding plane of the other half molecules.

The hydrogen-bonding interactions occur between the C—H in the CH_2_ group as donors and O in the salicylal moiety as acceptors (Table 1). These intermolecular hydrogen bonds construct an infinite ribbon. Interactions between the ribbons are van der Waals forces.

### Experimental

0.5 mmol of zinc oxide, 1 mmol of 4-methoxysalicylaldehyde and 1 mmol of (1*H*-pyrrol-2-yl)methanamine were dissolved in 10 ml methanol. After 3 ml ammonia was added, the result solution was then heated to 423 K for 12 h. The reactor was cooled to room temperature at a rate of 10 K h^-1^. The mixture was filtered and held at room temperature for 12 d. Colorless block crystals were isolated in 41% yield.

### Refinement

All H atoms were placed in geometrically idealized positions and constrained to ride on their parent atoms. *U*_iso_(H) values were set at 1.2 times *U*_eq_(C, N) for aromatic C and N in pyrrole ring groups and 1.5 times *U*_eq_(C) for CH_3_ groups.

### Crystal data

**Table:**

[Zn(C_13_H_13_N_2_O_2_)_2_]	*F*(000) = 1088
*M**_r_* = 523.88	*D*_x_ = 1.480 Mg m^−^^3^
Monoclinic, *C*2/*c*	Mo *K*α radiation, λ = 0.71073 Å
Hall symbol: -C 2yc	Cell parameters from 2301 reflections
*a* = 27.210 (4) Å	θ = 2.5–26.1°
*b* = 5.2239 (9) Å	µ = 1.09 mm^−^^1^
*c* = 24.335 (3) Å	*T* = 298 K
β = 137.179 (9)°	Block, colorless
*V* = 2351.1 (6) Å^3^	0.30 × 0.30 × 0.20 mm
*Z* = 4	

### Data collection

**Table:**

Bruker SMART APEX area-detector diffractometer	2881 independent reflections
Radiation source: fine-focus sealed tube	2429 reflections with *I* > 2σ(*I*)
graphite	*R*_int_ = 0.028
φ and ω scans	θ_max_ = 28.3°, θ_min_ = 2.2°
Absorption correction: multi-scan (*SADABS*; Sheldrick, 1996)	*h* = −36→35
*T*_min_ = 0.736, *T*_max_ = 0.812	*k* = −6→6
8100 measured reflections	*l* = −26→32

### Refinement

**Table:**

Refinement on *F*^2^	Primary atom site location: structure-invariant direct methods
Least-squares matrix: full	Secondary atom site location: difference Fourier map
*R*[*F*^2^ > 2σ(*F*^2^)] = 0.058	Hydrogen site location: inferred from neighbouring sites
*wR*(*F*^2^) = 0.154	H-atom parameters constrained
*S* = 1.08	*w* = 1/[σ^2^(*F*_o_^2^) + (0.0814*P*)^2^ + 2.3252*P*] where *P* = (*F*_o_^2^ + 2*F*_c_^2^)/3
2881 reflections	(Δ/σ)_max_ < 0.001
160 parameters	Δρ_max_ = 0.72 e Å^−^^3^
0 restraints	Δρ_min_ = −0.57 e Å^−^^3^

### Special details

**Table:**

Geometry. All e.s.d.'s (except the e.s.d. in the dihedral angle between two l.s. planes) are estimated using the full covariance matrix. The cell e.s.d.'s are taken into account individually in the estimation of e.s.d.'s in distances, angles and torsion angles; correlations between e.s.d.'s in cell parameters are only used when they are defined by crystal symmetry. An approximate (isotropic) treatment of cell e.s.d.'s is used for estimating e.s.d.'s involving l.s. planes.
Refinement. Refinement of *F*^2^ against ALL reflections. The weighted *R*-factor *wR* and goodness of fit *S* are based on *F*^2^, conventional *R*-factors *R* are based on *F*, with *F* set to zero for negative *F*^2^. The threshold expression of *F*^2^ > σ(*F*^2^) is used only for calculating *R*-factors(gt) *etc*. and is not relevant to the choice of reflections for refinement. *R*-factors based on *F*^2^ are statistically about twice as large as those based on *F*, and *R*- factors based on ALL data will be even larger.

### Fractional atomic coordinates and isotropic or equivalent isotropic displacement parameters (Å^2^)

**Table:**

	*x*	*y*	*z*	*U*_iso_*/*U*_eq_	
C1	0.93603 (16)	0.2495 (6)	0.29548 (18)	0.0401 (6)	
C2	0.87592 (16)	0.4163 (6)	0.24129 (18)	0.0398 (6)	
C3	0.81985 (18)	0.3941 (7)	0.2349 (2)	0.0503 (8)	
H3	0.7802	0.5026	0.1996	0.060*	
C4	0.82168 (18)	0.2211 (7)	0.2782 (2)	0.0550 (8)	
H4	0.7838	0.2105	0.2720	0.066*	
C5	0.8813 (2)	0.0602 (7)	0.3317 (2)	0.0490 (8)	
C6	0.93736 (19)	0.0707 (6)	0.3401 (2)	0.0462 (7)	
H6	0.9763	−0.0409	0.3754	0.055*	
C7	0.9380 (2)	−0.2809 (8)	0.4279 (2)	0.0673 (10)	
H7A	0.9385	−0.3953	0.3973	0.101*	
H7B	0.9317	−0.3774	0.4559	0.101*	
H7C	0.9830	−0.1896	0.4663	0.101*	
C8	0.86588 (16)	0.6031 (6)	0.19107 (18)	0.0418 (7)	
H8	0.8240	0.7009	0.1592	0.050*	
C9	0.88198 (18)	0.8391 (7)	0.1226 (2)	0.0507 (8)	
H9A	0.9208	0.9554	0.1449	0.061*	
H9B	0.8430	0.9389	0.1065	0.061*	
C10	0.85569 (18)	0.7069 (7)	0.0508 (2)	0.0523 (8)	
C11	0.8621 (2)	0.7591 (13)	0.0026 (3)	0.0938 (18)	
H11	0.8869	0.8941	0.0066	0.113*	
C12	0.8210 (3)	0.5539 (14)	−0.0582 (3)	0.103 (2)	
H12	0.8155	0.5318	−0.1004	0.123*	
C13	0.7934 (3)	0.4069 (13)	−0.0421 (3)	0.103 (2)	
H13	0.7646	0.2637	−0.0717	0.124*	
N1	0.90797 (13)	0.6538 (5)	0.18427 (15)	0.0398 (5)	
N2	0.81349 (18)	0.4967 (6)	0.02362 (19)	0.0571 (8)	
H2	0.8016	0.4320	0.0451	0.069*	
O1	0.99094 (12)	0.2514 (5)	0.30668 (15)	0.0552 (6)	
O2	0.88016 (16)	−0.1038 (6)	0.37455 (17)	0.0663 (7)	
Zn1	1.0000	0.46424 (10)	0.2500	0.0430 (2)	

### Atomic displacement parameters (Å^2^)

**Table:**

	*U*^11^	*U*^22^	*U*^33^	*U*^12^	*U*^13^	*U*^23^
C1	0.0412 (15)	0.0449 (16)	0.0473 (16)	−0.0004 (12)	0.0366 (14)	−0.0027 (13)
C2	0.0376 (14)	0.0469 (17)	0.0450 (16)	−0.0015 (12)	0.0334 (14)	−0.0038 (13)
C3	0.0426 (16)	0.065 (2)	0.0567 (19)	0.0051 (15)	0.0406 (16)	0.0019 (16)
C4	0.0501 (18)	0.073 (2)	0.065 (2)	−0.0037 (17)	0.0497 (18)	−0.0036 (18)
C5	0.058 (2)	0.0520 (18)	0.0553 (19)	−0.0101 (15)	0.0475 (18)	−0.0071 (15)
C6	0.0513 (18)	0.0469 (17)	0.0528 (18)	0.0015 (14)	0.0421 (16)	0.0016 (14)
C7	0.086 (3)	0.060 (2)	0.067 (2)	−0.011 (2)	0.059 (2)	0.0029 (19)
C8	0.0363 (14)	0.0465 (16)	0.0448 (16)	0.0037 (12)	0.0304 (14)	−0.0004 (13)
C9	0.0515 (18)	0.0459 (18)	0.060 (2)	−0.0014 (14)	0.0424 (17)	0.0043 (15)
C10	0.0449 (17)	0.067 (2)	0.0499 (18)	0.0130 (15)	0.0363 (16)	0.0157 (16)
C11	0.067 (3)	0.156 (5)	0.081 (3)	0.038 (3)	0.061 (3)	0.049 (3)
C12	0.081 (4)	0.168 (6)	0.059 (3)	0.047 (4)	0.051 (3)	0.013 (3)
C13	0.091 (4)	0.114 (5)	0.060 (3)	0.025 (3)	0.042 (3)	−0.013 (3)
N1	0.0355 (12)	0.0458 (13)	0.0423 (13)	−0.0016 (10)	0.0298 (11)	−0.0022 (11)
N2	0.0605 (19)	0.0552 (18)	0.0486 (16)	−0.0063 (13)	0.0378 (16)	−0.0074 (13)
O1	0.0481 (12)	0.0674 (15)	0.0712 (15)	0.0150 (11)	0.0504 (13)	0.0210 (12)
O2	0.0821 (18)	0.0695 (17)	0.0832 (19)	−0.0025 (15)	0.0719 (17)	0.0089 (15)
Zn1	0.0374 (3)	0.0513 (3)	0.0530 (3)	0.000	0.0372 (3)	0.000

### Geometric parameters (Å, °)

**Table:**

C1—O1	1.308 (3)	C9—N1	1.460 (4)
C1—C6	1.412 (4)	C9—C10	1.487 (5)
C1—C2	1.417 (4)	C9—H9A	0.9700
C2—C3	1.421 (4)	C9—H9B	0.9700
C2—C8	1.425 (4)	C10—C11	1.333 (5)
C3—C4	1.360 (5)	C10—N2	1.359 (5)
C3—H3	0.9300	C11—C12	1.471 (8)
C4—C5	1.391 (5)	C11—H11	0.9300
C4—H4	0.9300	C12—C13	1.318 (9)
C5—O2	1.367 (4)	C12—H12	0.9300
C5—C6	1.384 (5)	C13—N2	1.340 (6)
C6—H6	0.9300	C13—H13	0.9300
C7—O2	1.422 (5)	N1—Zn1	1.983 (2)
C7—H7A	0.9600	N2—H2	0.8600
C7—H7B	0.9600	O1—Zn1	1.923 (2)
C7—H7C	0.9600	Zn1—O1^i^	1.923 (2)
C8—N1	1.299 (4)	Zn1—N1^i^	1.983 (2)
C8—H8	0.9300		
			
O1—C1—C6	117.6 (3)	N1—C9—H9B	109.5
O1—C1—C2	123.5 (3)	C10—C9—H9B	109.5
C6—C1—C2	118.9 (3)	H9A—C9—H9B	108.1
C1—C2—C3	117.7 (3)	C11—C10—N2	110.3 (4)
C1—C2—C8	125.7 (3)	C11—C10—C9	132.7 (4)
C3—C2—C8	116.6 (3)	N2—C10—C9	116.9 (3)
C4—C3—C2	122.9 (3)	C10—C11—C12	104.0 (5)
C4—C3—H3	118.6	C10—C11—H11	128.0
C2—C3—H3	118.6	C12—C11—H11	128.0
C3—C4—C5	118.8 (3)	C13—C12—C11	107.7 (5)
C3—C4—H4	120.6	C13—C12—H12	126.1
C5—C4—H4	120.6	C11—C12—H12	126.1
O2—C5—C6	123.5 (3)	C12—C13—N2	109.2 (6)
O2—C5—C4	115.5 (3)	C12—C13—H13	125.4
C6—C5—C4	121.1 (3)	N2—C13—H13	125.4
C5—C6—C1	120.6 (3)	C8—N1—C9	117.3 (3)
C5—C6—H6	119.7	C8—N1—Zn1	120.3 (2)
C1—C6—H6	119.7	C9—N1—Zn1	122.2 (2)
O2—C7—H7A	109.5	C13—N2—C10	108.8 (5)
O2—C7—H7B	109.5	C13—N2—H2	125.6
H7A—C7—H7B	109.5	C10—N2—H2	125.6
O2—C7—H7C	109.5	C1—O1—Zn1	125.7 (2)
H7A—C7—H7C	109.5	C5—O2—C7	118.3 (3)
H7B—C7—H7C	109.5	O1—Zn1—O1^i^	109.36 (15)
N1—C8—C2	128.1 (3)	O1—Zn1—N1^i^	117.41 (10)
N1—C8—H8	116.0	O1^i^—Zn1—N1^i^	96.71 (10)
C2—C8—H8	116.0	O1—Zn1—N1	96.71 (10)
N1—C9—C10	110.7 (3)	O1^i^—Zn1—N1	117.41 (10)
N1—C9—H9A	109.5	N1^i^—Zn1—N1	120.10 (15)
C10—C9—H9A	109.5		
			
O1—C1—C2—C3	−180.0 (3)	C2—C8—N1—C9	174.4 (3)
C6—C1—C2—C3	0.1 (4)	C2—C8—N1—Zn1	−1.1 (4)
O1—C1—C2—C8	1.2 (5)	C10—C9—N1—C8	−102.9 (3)
C6—C1—C2—C8	−178.8 (3)	C10—C9—N1—Zn1	72.5 (3)
C1—C2—C3—C4	−0.1 (5)	C12—C13—N2—C10	−0.4 (6)
C8—C2—C3—C4	178.8 (3)	C11—C10—N2—C13	1.4 (5)
C2—C3—C4—C5	0.7 (5)	C9—C10—N2—C13	178.5 (4)
C3—C4—C5—O2	178.3 (3)	C6—C1—O1—Zn1	177.0 (2)
C3—C4—C5—C6	−1.3 (5)	C2—C1—O1—Zn1	−3.0 (5)
O2—C5—C6—C1	−178.3 (3)	C6—C5—O2—C7	−3.0 (5)
C4—C5—C6—C1	1.3 (5)	C4—C5—O2—C7	177.4 (3)
O1—C1—C6—C5	179.4 (3)	C1—O1—Zn1—O1^i^	−119.8 (3)
C2—C1—C6—C5	−0.7 (5)	C1—O1—Zn1—N1^i^	131.4 (3)
C1—C2—C8—N1	1.1 (5)	C1—O1—Zn1—N1	2.4 (3)
C3—C2—C8—N1	−177.8 (3)	C8—N1—Zn1—O1	−0.4 (3)
N1—C9—C10—C11	−139.4 (4)	C9—N1—Zn1—O1	−175.6 (2)
N1—C9—C10—N2	44.4 (4)	C8—N1—Zn1—O1^i^	115.5 (2)
N2—C10—C11—C12	−1.7 (4)	C9—N1—Zn1—O1^i^	−59.7 (3)
C9—C10—C11—C12	−178.1 (4)	C8—N1—Zn1—N1^i^	−127.6 (3)
C10—C11—C12—C13	1.4 (6)	C9—N1—Zn1—N1^i^	57.2 (2)
C11—C12—C13—N2	−0.6 (6)		

Symmetry codes: (i) −*x*+2, *y*, −*z*+1/2.

### Hydrogen-bond geometry (Å, °)

**Table:**

*D*—H···*A*	*D*—H	H···*A*	*D*···*A*	*D*—H···*A*
C9—H9A···O1^ii^	0.97	2.32	3.291 (4)	177

Symmetry codes: (ii) −*x*+2, *y*+1, −*z*+1/2.
